# Chain mediation of sports cognition and moderate to vigorous physical activity in the socioeconomic status-physical quality relationship among Macao adults

**DOI:** 10.3389/fpsyg.2025.1658377

**Published:** 2026-01-16

**Authors:** Xuehui Zhang, Yibo Gao, Mingzhe Li, Jin He, Xiang Pan, Lupei Jiang, Koya Suzuki, Yanfeng Zhang

**Affiliations:** 1 College of Sports Science, Hefei Normal University, Hefei, China; 2 China Institute of Sport Science, Beijing, China; 3 Graduate School of Health and Sports Science, Juntendo University, Inzai, Japan

**Keywords:** Macao adults, socioeconomic status, physical quality, sports cognition, moderate to vigorous physical activity, chain mediation

## Abstract

**Objective:**

This study aimed to investigate the relationship between socioeconomic status (SES) and physical quality (PQ) among adults, with a particular focus on the chain-mediated effects of sports cognition (SC) and moderate to vigorous physical activity (MVPA).

**Methods:**

Based on data from the 2020 Macao Special Administrative Region Citizen’s Physical Fitness and Health Surveillance, a total of 3,085 adults aged 19–59 years were analyzed using SPSS 29.0 and AMOS 29.0.

**Results:**

The results showed that (1) SES was positively associated with PQ (*p* < 0.01); (2) SES exerted a significant positive direct effect on PQ (β = 0.198, 95% CI: [0.004, 0.400]) and a significant total indirect effect (β = 0.148, 95% CI: [0.099, 0.195]). Specifically, SES indirectly affected PQ through SC (β = 0.091, 95% CI: [0.056, 0.132]), MVPA (β = 0.047, 95% CI: [0.020, 0.077]), and the sequential SC-MVPA pathway (β = 0.010, 95% CI: [0.005, 0.015]), accounting for 26.30%, 13.58%, and 2.89% of the total effect, respectively.

**Conclusions:**

These findings suggest that SES is associated with adult PQ through both individual and chain-mediated pathways, providing correlational evidence that may inform the development of future intervention strategies to enhance PQ among adults in Macao. Moreover, the results underscore the crucial role of cognitive enhancement in promoting physical health.

## 1 Introduction

Physical Quality (PQ) is a crucial indicator that reflects the physical condition of the human body and serves as a fundamental basis for individual survival, development, and long-term health. It is also a prerequisite for maintaining overall wellbeing. However, numerous global studies have reported a declining trend in the overall PQ levels of the population. This trend is mainly reflected in the rise of body mass index (Fugiel et al., 2022), reduced cardiorespiratory endurance (Doyon et al., 2021), and decreased flexibility (Basterfield et al., 2022), among other indicators. The general deterioration in PQ has become a key contributing factor to increasing mortality rates and the growing prevalence of chronic non-communicable diseases (Strandberg et al., 2025). As a result, healthcare expenditures have surged dramatically, placing a mounting burden on national public health systems. In light of this global health crisis, urgent action is needed across all sectors of society to raise awareness and implement effective interventions.

The family serves not only as the primary context of adults’ daily lives but also as a key manifestation of socioeconomic status (SES). As a comprehensive indicator of household resources and social standing (Sager La Ganga et al., 2025), SES has been consistently linked to a wide range of health-related outcomes (Qin et al., 2025; Sánchez-Díaz et al., 2025). Previous studies have demonstrated that family-level factors, including resource allocation, educational attainment, and lifestyle patterns, play an important role in shaping individuals’ PQ levels (Nikolakaros et al., 2020). Based on this, this study proposed the first hypothesis that SES has a positive predictive effect on PQ.

Sports cognition (SC) (Fetter et al., 2019) refers to an individual’s cognition of the health benefits, challenges, methods, and both personal and social values related to physical activity participation. It encompasses both knowledge-based and attitude-based. Existing studies have confirmed that both SC (Ntoumanis et al., 2021; Sevild et al., 2022) and Moderate to Vigorous Physical Activity (MVPA) (Yerrakalva et al., 2024; Bailey et al., 2025) are positively associated with PQ. Moreover, research has found that perceptions of exercise benefits and health-related knowledge—such as understanding health-enhancing mechanisms and possessing self-efficacy—serve as key psychological resources that promote physical activity (Annesi, 2022; Petzold et al., 2024). Importantly, higher levels of SC are theoretically expected to lead to higher levels of MVPA. According to the cognition–attitude–behavior framework, enhanced exercise-related cognition improves attitudes, motivation, and self-efficacy toward physical activity, which subsequently translate into increased behavioral engagement in MVPA. Thus, SC is not only a direct psychological predictor of PQ but also an antecedent of MVPA, providing a rationale for testing a sequential (chain) mediation pathway rather than parallel mediation alone. Accordingly, the following hypotheses were proposed. Enhancing such perceptions (e.g., believing that exercise prevents chronic diseases and improves PQ) and increasing self-efficacy are considered critical pathways for fostering long-term physical activity habits. Accordingly, the following hypotheses were proposed: (2) SC mediates the relationship between SES and PQ; and (3) MVPA mediates the relationship between SES and PQ.

Although existing studies have separately examined the bivariate relationships among SES, SC, MVPA, and PQ, few have integrated these four variables into a unified analytical framework to systematically test the chain mediating roles of SC and MVPA in the relationship between SES and PQ in adults. In order to address this research gap and build upon the aforementioned theoretical foundation, this study formulates a fourth hypothesis: that SC and MVPA jointly constitute a chain mediation mechanism in the pathway linking SES and adults’ PQ. This hypothesis aims to provide a deeper understanding of how SES exerts long-term effects on the PQ of the adult population.

Macao is a region within China that is distinguished by relatively advanced socio-economic development and compact urban-rural spatial distribution. The residents of these areas have been observed to lead fast-paced lives, with high residential density (He et al., 2023) and limited opportunities for physical activity. Despite its position as one of the world’s highest ranking regions in terms of per capita GDP (Li et al., 2019), research findings suggest that the adult population of Macao exhibits a decline in PQ (Choi et al., 2025) is becoming increasingly evident. Thus, giving rise to a well-established phenomenon that has come to be recognized as “economic affluence-related health risks.” Furthermore, Macau exhibits significant socioeconomic disparities among its population, with uneven distribution of education and occupational type (Li et al., 2025). This presents a unique context for examining how household SES influences individual physical fitness.

**FIGURE 1:**
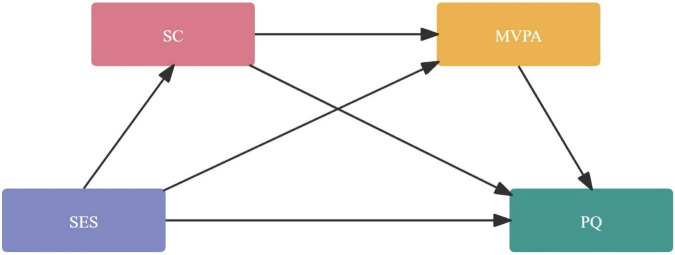
Relationship between SES, SC, MVPA, and PQ. SES is Socioeconomic Status, SC is Sports Cognition, MVPA is Moderate to Vigorous Physical Activity, and PQ is Physical Quality.

Building upon the aforementioned theoretical framework and the unique social context of Macao, this study aims to systematically examine the relationships among SES, SC, MVPA, and PQ in the adult population of Macao. By integrating these constructs into a single conceptual model and evaluating their sequential mediation pathways, this study advances previous research that has typically examined them only in isolation or in pairs. Specifically, the study aims to: (1) to examine the direct effects of SES on adult PQ levels; (2) to assess the mediating effects of SC and MVPA between SES and PQ separately; and (3) to examine the chain mediating effects of SC-MVPA between SES and PQ. By constructing a comprehensive theoretical model to reveal the potential pathways through which SES affects adult PQ, this study aims to provide a theoretical basis and policy recommendations for improving adult fitness levels and promoting health equity (Figure 1).

## 2 Materials and methods

### 2.1 Subjects

The data used in this study were drawn from the 2020 Physical Quality Surveillance Data of the Macao Special Administrative Region, targeting adults aged 19–59 years. The survey was conducted between July 1 and November 30, 2020. Due to the impact of the COVID-19 pandemic, stratified random cluster sampling was adopted to ensure representativeness. For sampling purposes, Macao’s seven administrative districts were divided into three strata. Adult participants were selected from both government agencies and private organizations using whole-group (department-level) cluster sampling. One-third of the participants were recruited from government institutions, while the remaining two-thirds were drawn from private-sector organizations. To ensure a balanced representation across different age groups, resampling was conducted to maintain an even age distribution within each stratum.

Inclusion criteria for the surveillance subjects were as follows: individuals had to be in good physical health, without congenital or hereditary diseases, and free from any acute or chronic illnesses. Participants were also required to possess basic self-care abilities and language skills, and to be capable of performing simple physical activities. A total of 3,860 adults were surveyed.

### 2.2 Selection of indicators

#### 2.2.1 Socio-economic status of households

SES is typically assessed using three indicators: educational attainment, income level, and occupational type (Korous et al., 2018; Wang et al., 2022). However, in consideration of the sensitivity surrounding the disclosure of economic income information among individuals in the Macao region and the principle of privacy protection, this study did not conduct a survey of participants’ income. However, previous studies have adopted a two-dimensional approach for SES measurement (Oliveira et al., 2025; Yu et al., 2025). Following this approach, the present study used education level and occupational type to construct the SES indicator.

Education level was categorized into four levels: (1) elementary school and below, (2) secondary and middle school, (3) undergraduate, and (4) postgraduate degree and above. Occupational type was divided into five levels: (1) casual workers, unemployed individuals, unskilled laborers, and general labor class; (2) manual workers and self-employed persons; (3) general management and general professional or technical staff; (4) middle management and mid-level professional or technical staff; and (5) senior managers and senior professional or technical staff.

Before conducting principal component analysis (PCA), both indicators were standardized using Z-scores. The Kaiser-Meyer-Olkin (KMO) value was 0.706, and Bartlett’s test of sphericity yielded χ^2^ = 351.619 (*p* < 0.01), indicating suitability for PCA. One principal component was extracted (eigenvalue = 1.148), explaining 63.843% of the total variance. The factor loadings for education level and occupational type were 0.811 and 0.634, respectively. The final SES score was computed as follows: SES = (0.811 * Z_*education*_ + 0.634 *Z_*occupation*_)/1. 148. Higher scores represent a higher level of SES.

#### 2.2.2 Physical quality

PQ was assessed using four physical fitness indicators collected during the monitoring process: grip strength, seated forward bending, one-legged stance with eyes closed, and choice reaction time, representing muscular strength, flexibility, balance, and sensitivity (neural responsiveness), respectively. The scoring criteria were based on Instituto do Desporto do Governo da Regiao Administrativa Especial de Macau Sports Bureau of Macao SAR Government (2018) for adults aged 20 to 59. Each indicator was converted to a five-point scale, and a higher total score indicates better PQ.

#### 2.2.3 Sports cognition

SC consists of two core dimensions. The first is knowledge-based cognition, which refers to an individual’s objective understanding of exercise, such as appropriate modalities, recommended frequency and intensity, and the associated health benefits. The second is attitude-based cognition, which reflects an individual’s subjective orientation toward exercise, including their value judgments, emotional stance, and willingness to engage in physical activity (Balla et al., 2024). These cognitive aspects not only shape attitudes toward physical activity but also directly influence behavioral intentions and the likelihood of sustained engagement (Chen et al., 2024).

SC was measured using 11 items from the corresponding section of the questionnaire. The reliability and validity of this section were examined in the present study. The sport cognition items and options are shown in Table 1.

**TABLE 1:** Sport cognition entries and options.

Questioners	Options
Sport is a positive and uplifting activity.	1 = Strongly disagree 2 = Disagree 3 = Neutral 4 = Agree 5 = Strongly agree
Sport is beneficial for maintaining good health.	
Sport can help relieve anxiety and irritability.	
Sport can strengthen a person’s willpower.	
Sport is boring.	
Sport is a highly worthwhile activity.	
Sport is a part of my life that I can do without.	
No matter how busy I am, I should make time for sport.	
The cost of sport is worth it.	
I am willing to persuade people around me to sport with me.	
The Sport skills I have are scientific and correct.	

An exploratory factor analysis (EFA) yielded a Kaiser-Meyer-Olkin (KMO) value of 0.786, and Bartlett’s test of sphericity produced χ^2^ = 624.86 (*p* < 0.001). Based on eigenvalues greater than 1 and multiple rounds of rotation, three latent factors were extracted, explaining a total of 84.969% of the variance. Factor loadings ranged from 0.605 to 0.841.

A confirmatory factor analysis (CFA) was subsequently conducted. Using the model fit criteria recommended by Steiger (1990) (Carmines and McIver, 1983), the following fit indices were obtained: χ^2^/df = 1.964 (<5.000), RMSEA = 0.032 (<0.080), GFI = 0.812 (>0.800), CFI = 0.835 (>0.800), and IFI = 0.901 (>0.800), all of which indicated good model fit and structural stability (Jebb et al., 2021; Steiger, 1990). Reliability analysis showed that the overall Cronbach’s alpha coefficient was 0.872. The Cronbach’s alpha values for the two first-order latent constructs were 0.914 and 0.883, respectively. These results demonstrate that the 11 items effectively assess individuals’ levels of sports-related cognition and motivation.

#### 2.2.4 MVPA

Moderate to Vigorous Physical Activity (MVPA) was assessed using the International Physical Activity Questionnaire—Short Form (IPAQ-SF), a widely used and validated instrument. The scale has demonstrated good test-retest reliability (Spearman’s ρ≈ 0.80) and acceptable construct validity, with a median correlation coefficient of approximately 0.30 with objective measures (Craig et al., 2003).

#### 2.2.5 Control variables

Based on existing research, the following demographic variables were included as control variables: gender, age, and years of residence. These are commonly used covariates in studies examining physical activity and PQ.

### 2.3 Survey procedures

Before data collection, interviewers received training to ensure a thorough understanding of the survey’s objectives and procedures. Respondent information was obtained from the Statistics and Census Service of the Macao SAR, and stratified sampling was conducted accordingly. Data were collected through face-to-face household interviews. During each session, interviewers explained the purpose of the study. Written informed consent was obtained from all participants before survey administration. After data collection, participants’ personal information was anonymized using three-level coding to ensure privacy protection. Ethical approval for this study was granted by the China Institute of Sports Science (Approval No.: CISSLA-20190607).

### 2.4 Statistical methods

All statistical analyses were performed using SPSS 29.0 and AMOS 29.0. The Kolmogorov–Smirnov (K–S) test was used to assess the normality of continuous variables.

Descriptive statistics for continuous variables were expressed as mean ± standard deviation (M ± SD), and categorical variables were presented as frequencies and percentages (n,%). The K–S test results indicated that the data followed a normal distribution. Accordingly, independent samples *t*-tests were used for continuous variables, and chi-square (χ^2^) tests were applied to categorical variables.

To examine relationships among key variables—SES, SC, MVPA, and PQ—Pearson correlation analysis was conducted. Multiple linear regression analysis was employed to assess the effects of SES, SC, and MVPA on PQ.

Mediation analysis was conducted using the PROCESS macro in SPSS. The Bootstrap method with 5,000 resamples was used to construct 95% CI for indirect effects. If the confidence interval did not include 0, the mediation effect was considered statistically significant. A two-tailed *p* < 0.05 was regarded as indicating statistical significance.

## 3 Results

To test for potential common method bias, Harman’s one-factor test was performed. The results showed that three factors had eigenvalues greater than 1, with the first factor explaining 31.26% of the total variance. As this is below the critical threshold of 40%, common method bias was not a significant concern in this study (Podsakoff et al., 2003). Multivariate linear regression analysis was conducted with PQ scores as the dependent variable. The variance inflation factors (VIF) ranged from 1.033 to 1.568, all significantly below 10, indicating no multicollinearity issues within the model. The model demonstrated overall satisfactory fit, consistent with the assumptions of multivariate regression analysis.

### 3.1 Descriptive statistics

After excluding invalid samples due to missing physical quality test data or incomplete questionnaire responses, the final analytical sample comprised 3,058 participants, yielding an effective data rate of 79.2%. Among them, 1,902 (62.2%) were aged 19–29 years and 1,156 (37.8%) were aged 30–59 years. Significant differences were observed between the two age groups in gender, educational attainment, occupational category (all *p* < 0.001). The overall sample consisted of 1,148 males (37.5%) and 1,910 females (62.5%), with a higher proportion of females in the younger group. Younger adults showed higher SES and SC scores but lower MVPA and PQ levels compared with middle-aged adults (all *p* < 0.001). The mean SES, SC, MVPA, and PQ scores for the total sample were 2.15, 30.35, 190.36 min/week, and 19.22, respectively, with an average residence duration of 29.53 years (Table 2).

**TABLE 2:** Basic information (*n* = 3,058).

Variables	19–29 years	30–59 years	19–59 years	Statistical magnitude	*P*
Gender, n(%)				22.821	< 0.001
Male	652 (34.3)	496 (42.9)	1,148 (37.5)		
Female	1 250 (65.7)	660 (57.1)	1,910 (62.5)		
Educational level, n(%)				427.148	< 0.001
Primary school and below	4 (0.2)	74 (6.4)	78 (2.6)		
Secondary school	178 (9.4)	380 (32.9)	558 (18.2)		
Bachelor’s degree	1 433 (75.3)	524 (45.3)	1,957 (64.0)		
Master’s degree or above	287 (15.1)	178 (15.4)	465 (15.2)		
Occupation, n (%)				81.692	< 0.001
Temporary workers, unemployed, or unskilled laborers	288 (15.1)	252 (21.8)	540 (17.7)		
Manual workers and self-employed persons	30 (1.6)	66 (5.7)	96 (3.1)		
General management versus general professional and technical staff	572 (30.1)	332 (28.7)	904 (29.6)		
Middle management and middle-level professional and technical staff	724 (38.1)	318 (27.5)	1,042 (34.1)		
Senior managers and senior professional and technical staff	288 (15.1)	188 (16.3)	476 (15.6)		
SES (scores), Mean ± SD	2.19 ± 0.54	2.10 ± 0.56	2.15 ± 0.55	4.866	< 0.001
SC (scores), Mean ± SD	30.50 ± 4.67	30.15 ± 5.15	30.35 ± 4.89	2.120	< 0.001
MVPA (week/min), Mean ± SD	174.48 ± 144.97	205.63 ± 165.97	190.36 ± 154.77	–3.604	< 0.001
PQ (scores) Mean ± SD	19.00 ± 3.50	19.52 ± 3.33	19.22 ± 3.44	–4.528	< 0.001
Years of residence (years), Mean ± SD	24.03 ± 10.51	38.59 ± 14.81	29.53 ± 14.19	–29.275	< 0.001
Total, n(%)	1,902 (62.2)	1,156 (37.8)	3,058		

Years of residence: *t*-test for independent samples, t; chi-square test for all other tests, χ^2^.

### 3.2 Correlation analysis

Correlation analysis (Table 3) revealed that SES was weakly positively correlated with SC (*r* = 0.097, *p* < 0.01), MVPA (*r* = 0.077, *p* < 0.01), and PQ (*r* = 0.055, *p* < 0.01). SC was weakly positively associated with both MVPA (*r* = 0.133, *p* < 0.01) and PQ (*r* = 0.172, *p* < 0.01), and MVPA also showed a weak positive correlation with PQ (*r* = 0.145, *p* < 0.01). Among the control variables, gender was weakly negatively correlated with SES, SC, and MVPA (*p* < 0.01), but showed no significant correlation with PQ. Age and years of residence were weakly negatively associated with SES and SC (*p* < 0.01), but weakly positively correlated with MVPA and PQ (*p* < 0.01).

**TABLE 3:** Correlation between variables (*n* = 3,058).

Variables	SES	SC	MVPA	PQ	Gender	Age	Years of residence
SES	1						
SC	0.097**	1					
MVPA	0.077**	0.133**	1				
PQ	0.055**	0.172**	0.145**	1			
Gender	-0.087**	-0.116**	-0.122**	0.005	1		
Age	-0.107**	-0.149**	0.064**	0.080**	-0.028	1	
Years of residence	-0.098**	-0.047**	0.048**	0.046**	-0.045**	0.581**	1

**P < 0.01.

### 3.3 Mediation effects analysis

Stepwise multiple linear regression was used to examine the predictive effects of SES on PQ, with SC and MVPA as mediators, and gender, age, and years of residence as control variables. In Model 1, only age (β = 0.081, *p* < 0.01) showed a significant positive association with PQ, while gender and years of residence were not significantly related. In Model 2, after including SES, the pattern remained consistent with Model 1, and SES also demonstrated a significant positive effect on PQ (β = 0.066, *p* < 0.01). In Model 3, after further adding SC, gender (β = 0.036, *p* < 0.01) and age (β = 0.121, *p* < 0.01) were both positively associated with PQ. SES (β = 0.053, *p* < 0.01) and SC (β = 0.189, *p* < 0.05) also had significant positive effects. In Model 4, MVPA was added to the regression. The effects of the control variables remained similar to Model 3. Age (β = 0.047, *p* < 0.01) remained a significant predictor. Additionally, SES (β = 0.111, *p* < 0.01), SC (β = 0.045, *p* < 0.01), and MVPA (β = 0.174, *p* < 0.01) all showed significant positive associations with PQ—indicating that higher levels of these factors corresponded to better PQ (Table 4).

**TABLE 4:** Regression results of chain mediation effect (*n* = 3,058).

Variables	Model 1			Model 2			Model 3			Model 4		
	*b*	SE	β	*b*	SE	β	*b*	SE	β	*b*	SE	β
Gender	0.051	0.118	0.007	0.095	0.118	0.013	0.252	0.117	0.036**	0.336	0.117	0.047**
Age	0.025	0.006	0.081**	0.027	0.006	0.086**	0.038	0.006	0.121**	0.035	0.006	0.111**
Years of residence	0.001	0.005	-0.001	0.001	0.005	0.003	-0.002	0.005	-0.008	-0.002	0.005	-0.009
SES				0.414	0.105	0.066**	0.329	0.104	0.053**	0.283	0.103	0.045**
SC							0.296	0.026	0.189*	0.272	0.026	0.174**
MVPA										0.002	0.000	0.117**
*F*	7.842			9.787			33.539			34.740		
*R* ^2^	0.006			0.011			0.045			0.058		

**P < 0.01; *P < 0.05.

**FIGURE 2:**
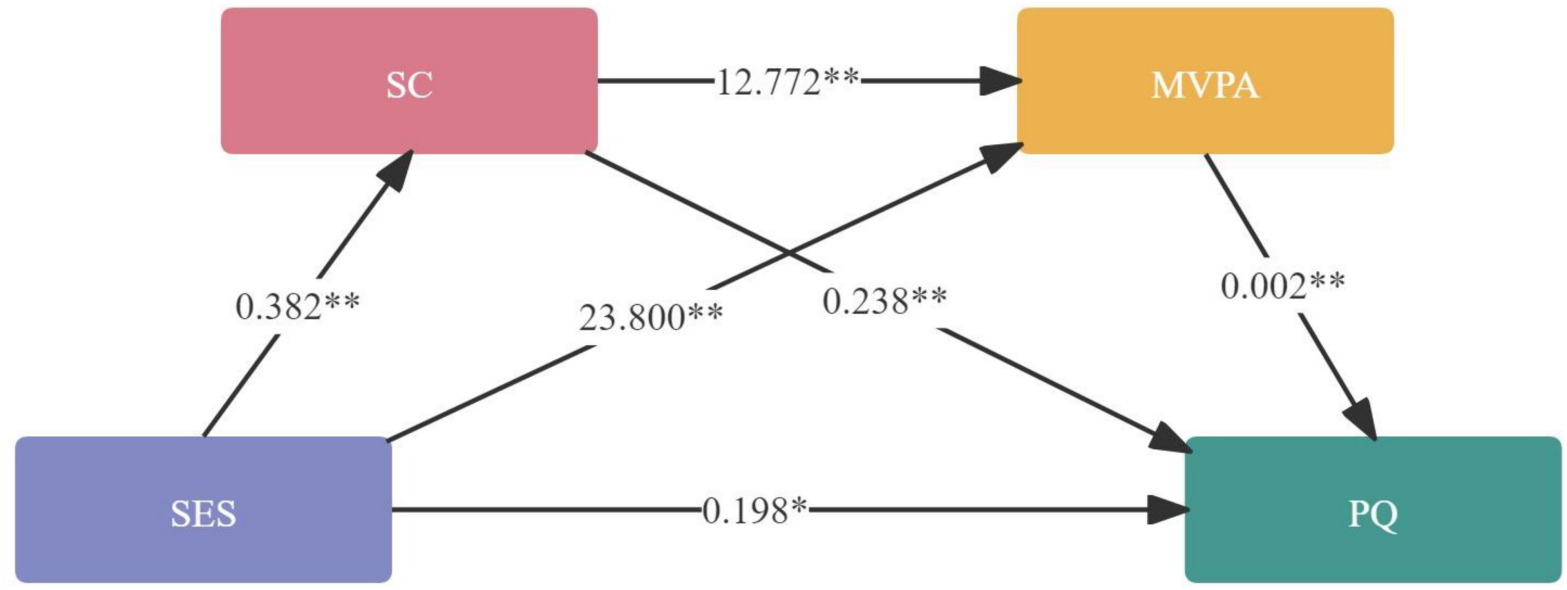
Chain mediation effect. SES, socioeconomic status; MS, motor skills; MVPA, moderate to vigorous physical activity; PF, physical fitness. The numbers currently shown between variables are direct path coefficients into MVPA. Sport cognition entries and options. *P < 0.05, **P < 0.01.

The mediation results are presented in Table 5 and Figure 2. The indirect effects of SES on PQ were statistically significant, as the 95% CI for all mediation paths did not include zero. This indicates that both SC and MVPA played significant mediating roles in the relationship between SES and PQ. Specifically, the first indirect effect followed the pathway SES → SC → PQ, with an effect estimate of 0.091 and a 95% CI of (0.056, 0.132), accounting for 26.30% of the total effect. The second indirect effect, along the pathway SES → MVPA → PQ, yielded an effect estimate of 0.047 with a 95% CI of (0.020, 0.077), representing 13.58% of the total effect. The third indirect effect, representing the chain mediation pathway SES → SC → MVPA → PQ, had an effect estimate of 0.010 with a 95% CI of (0.0005, 0.015), contributing 2.89% to the total effect. These results collectively indicated that SES was associated with adult PQ not only through the independent mediating effects of SC and MVPA but also via a sequential pathway involving both variables. The detailed results of the chain mediation effect path analysis are presented in Table 6.

**TABLE 5:** Standardized direct and indirect pathways (*n* = 3,058).

Pathways	Effect	BootSE^1^	95%CI		Contribution rate (%)
			LLCI	ULCI	
Total effect	0.346	0.104	0.141	0.55	100.00%
Direct effect	0.198	0.103	0.004	0.400	57.23%
Total indirect effect	0.148	0.025	0.099	0.195	42.77%
SES-SC-PQ	0.091	0.018	0.056	0.132	26.30%
SES-MVPA-PQ	0.047	0.015	0.020	0.077	13.58%
SES-SC-MVPA-PQ	0.010	0.002	0.005	0.015	2.89%

^1^5,000 Bootstrap samples.

**TABLE 6:** Chain mediation path analysis (*n* = 3,058).

Variables	Model 1			Model 2			Model 3		
	*b*	SE	β	*b*	SE	β	*b*	SE	β
SES-	0.382	0.0666	0.095**	23.800	6.4044	0.085**	0.1980	0.1010	0.032*
SC				12.771	1.6003	0.182**	0.2380	0.0259	0.153**
MVPA							0.0020	0.0003	0.090**
*F*	32.9589			41.9683			57.0674		
*R* ^2^	0.0091			0.022			0.0457		

**P < 0.01; *P < 0.05.

To further examine the association between SES and PQ, stratified regression analyses were conducted by age group and gender, adjusting for potential confounders. The results indicated that SES was positively associated with PQ across most subgroups. Specifically, the association was significant in the 19–29 age group (β = 0.049, *p* < 0.05) and in the 30–59 age group (β = 0.079, *p* < 0.01). Regarding gender, the association was significant among females (β = 0.062, *p* < 0.01), whereas it was not statistically significant among males (β = 0.045, *p* > 0.05). These findings suggest that the positive effect of SES on physical fitness may vary by age and gender (Table 7).

**TABLE 7:** Stratified analysis (*n* = 3,058).

Groups	*b*	SE	β
19–29 Years	0.315	0.144	0.049*
30–59 Years	0.472	0.151	0.079**
Male	0.306	0.182	0.045
Female	0.374	0.127	0.062**

b, unstandardized coefficient; SE, Standard Error; β, Standardized Regression Coefficient; *p < 0.01; *p < 0.05.

## 4 Discussion

The ethnic composition, geographic characteristics, economic structure, and lifestyle patterns in Macao differ significantly from those in Mainland China, and these contextual factors inevitably exert diverse influences on health outcomes (Jiang et al., 2025). Against this backdrop, the present study examined the relationships among SES, SC, MVPA, and PQ in Macao adults. A chain mediation model was constructed, revealing multiple indirect pathways through which SES was associated with PQ.

### 4.1 The direct effect of socioeconomic status on physical quality

SES emerged as a significant positive predictor of PQ, with a direct effect accounting for 42.77% of the total effect. This highlights the pivotal role of SES in shaping individual PQ. Households with higher SES tend to enjoy a range of advantages over lower-SES households. Firstly, they typically reside in neighborhoods characterized by a well-developed built environment and greater access to fitness infrastructure, such as public sports facilities and recreational spaces (McGrath et al., 2016). These external resources not only make physical activity more feasible but also contribute directly to improvements in PQ levels (McGrath et al., 2016). In addition, high-SES communities often foster a more health-conscious social environment. Residents are more likely to encounter positive social norms related to fitness, which can subtly influence and reinforce individual exercise behaviors. Furthermore, individuals from higher-SES households are usually better educated, enabling them to better understand the benefits of exercise for physical health and quality of life. This often leads to greater motivation to engage in and maintain regular physical activity. Moreover, individuals from higher-SES households face fewer financial constraints when it comes to accessing exercise facilities, equipment, or health services (Rodrigues et al., 2018). This economic advantage lowers the practical barriers to initiating and sustaining regular physical activity. Additionally, time management is another factor that should not be overlooked. Individuals from higher-SES backgrounds are often better able to plan their daily schedules and make efficient use of fragmented leisure time (Dunton et al., 2020), transforming these intervals into opportunities for physical activity and, ultimately, achieving sustained gains in PQ.

Macao’s high population density and the concentration of community facilities make environmental accessibility a pivotal factor in health disparities related to SES (Lei et al., 2023). Households in the higher SES bracket are more likely to live in neighborhoods with safer streets and more accessible recreational spaces, whereas those in the lower bracket face barriers to physical activity (Zhou et al., 2013). Compared to mainland China, Macao’s compact urban layout and higher level of educational attainment further encourage healthy behaviors among higher SES groups (Wong et al., 2017). Exposure to diverse health concepts also encourages participation in structured PA. These findings suggest that SES promotes mental health through multiple pathways and indicate that interventions should focus on improving equitable access to supportive environments and health resources.

Another consideration relates to the operationalization of SES. Because income was not available in the dataset, SES was measured using only education and occupation. While these indicators are commonly used, excluding income may have limited our ability to fully capture the socioeconomic gradient and may have slightly biased the strength of the associations observed. Future studies should incorporate income or use composite SES measures to enhance the precision of socioeconomic assessment. In sum, SES was associated with the development and maintenance of adult PQ through a multidimensional mechanism involving the built environment, sociocultural norms, health literacy, financial capacity, and time management.

### 4.2 Mediation effects of sports cognition

As discussed earlier, individuals with higher SES tend to have more comprehensive knowledge of the link between exercise and physical health. This is partly because higher-SES households generally enjoy broader access to health education resources and reliable health information (Bukman et al., 2014), which helps members build a more systematic understanding of the benefits of exercise. These individuals often have higher educational attainment and stronger health awareness, making them more inclined to view physical activity as an essential component of a healthy lifestyle and PQ. As a result, they are more likely to develop positive attitudes toward exercise at the cognitive level (Pampel et al., 2010). Therefore, SES can significantly enhance individuals’ SC by improving their health identity and awareness of physical literacy.

Once these internalized positive attitudes are formed, individuals are more likely to participate in physical activity autonomously, even in the absence of external pressure or supervision. This internal motivation strengthens their consistency in physical activity participation (Teixeira et al., 2012), which in turn contributes to improvements in PQ. These findings support the study’s hypothesis that SC mediates the relationship between SES and PQ.

### 4.3 Mediation effects of moderate to high intensity physical activity

Research has shown that individuals from lower SES groups experience significantly higher rates of chronic non-communicable diseases, such as early-onset hypertension (Leng et al., 2015), chronic respiratory conditions (Gershon et al., 2012), and cardiovascular diseases (Rosengren et al., 2019). This disparity is often attributed to heightened life stress and the absence of sustained healthy lifestyle habits. In contrast, individuals from higher SES households typically demonstrate stronger exercise awareness and benefit from more stable economic conditions. These advantages allow them to allocate more time and resources toward engaging in MVPA (Bauman et al., 2012). As a result, higher SES can lead to improved MVPA participation, which in turn contributes to enhanced PQ. The improvement in MVPA levels is not an isolated behavioral outcome, but rather occurs through a structured and cumulative process facilitated by participation in organized sports programs. These programs provide individuals with repeated opportunities to develop foundational motor skills, which enhance their physical competence and self-efficacy (Robinson et al., 2015). In turn, this skill acquisition fosters more sustained engagement in physical activity and drives improvements in PQ. In other words, MVPA promotes PQ through a broader chain mediation mechanism: sports participation → improvement in basic physical skills → enhancement in PQ. This mediating pathway supports the study’s hypothesis that MVPA serves as a significant mediator in the relationship between SES and PQ.

### 4.4 Chain mediation effects of sports cognition and moderate-to-high intensity physical activity

The chain mediation model proposed in this study SES → SC → MVPA → PQ demonstrates the pivotal role of SC and MVPA in linking household SES to PQ among Macao adults. The development of SC often originates from the activation of individual motivation (Albuquerque et al., 2019). According to the Social-Ecological Theory of Maximization (Chaves et al., 2020), individuals tend to make behavioral choices that maximize perceived benefits within their environmental context. For adults, motivational maturity is not only reflected in responsiveness to external cues but also in a stronger sense of autonomy, proactive engagement, and long-term behavioral persistence Once health-related motivation is activated, individuals are significantly more likely to engage in MVPA (Teixeira et al., 2012).

Furthermore, external motivation can gradually be internalized into intrinsic motivation, where individuals engage in physical activity out of personal interest, enjoyment, or a sense of identity with sport (Chame et al., 2019). This process may enhance attitudes toward physical activity and support the acquisition and maintenance of exercise behaviors over time. As such, increased self-efficacy could theoretically help individuals set activity goals, adhere to exercise regimens, and show greater resilience when facing obstacles (Trost et al., 2003; Peterson et al., 2013), which are important for promoting higher MVPA levels and, ultimately, improving PQ. These statements are offered as theoretical interpretations for contextual understanding and were not directly measured in this study.

Additionally, the explanatory power of the models was modest, with R^2^ values reaching only 0.058. However, small R^2^ values are common in social and behavioral research, particularly when predicting complex constructs influenced by multiple distal and proximal factors. Previous studies have emphasized that low coefficients of determination do not necessarily undermine the theoretical relevance of the findings, as meaningful effects in population-level behavioral research often manifest as small effect sizes.

In sum, internal and external motivations interact and reinforce one another, creating both a supportive environment and a sustained internal drive for enhancing PQ. As discussed in previous sections, SES positively correlates SC, and MVPA significantly contributes to PQ. The present study hypothesizes that SES exerts an indirect promote PQ through SC and MVPA. SC (knowledge and attitude dimensions) partially mediates this process (accounting for approximately 2.89% of the total effect), suggesting that while cognitive enhancement is not the primary pathway, it remains a crucial component in the formation of healthy behaviors.

### 4.5 Limitations

This study has several limitations. First, although the large sample size (*n* = 3,058) yielded statistically significant results, the correlation coefficients were generally weak in magnitude (as shown) in Table 3. This indicates that the practical significance and predictive value of these associations are limited. Second, the measurement of MVPA in this study was based on a self-reported questionnaire, which may be subject to recall bias. In future research, more accurate assessments can be achieved by combining calibrated questionnaires with objective measures such as accelerometers. Third, this study utilized data from the 2020 Macao Special Administrative Region Citizen’s Physical Fitness and Health Surveillance, and the control variables included were limited. Other potentially influential variables were not accounted for, which may have introduced confounding effects. Future studies could employ in-depth small-scale sampling to incorporate a broader set of control variables and reduce bias. Finally, the age range of participants in this study (19–59 years) spans multiple stages of adulthood. Given the behavioral and cognitive shifts that occur as individuals transition from youth to middle age, future research should consider stratifying age groups more precisely to enhance the generalizability of the findings.

## 5 Conclusion

The present study identified a chain-mediated mechanism through which SES was associated with PQ via SC and MVPA among adults in Macao. Higher SES was found to be associated with stronger SC, which in turn promoted MVPA participation and subsequently enhanced PQ. The findings emphasize the significance of enhancing SC and PA support among individuals with low SES, with the objective of promoting equitable access to PA resources and reducing disparities in PQ.

## Data Availability

The datasets presented in this article are not readily available because the data are not publicly available due to privacy. Requests to access the datasets should be directed to zhangyanfeng0310@126.com.
